# Implementing an Online Sexually Transmitted Infection Testing Service for Young People in Regional and Rural Victoria, Australia: Insights From Local Public Health Authorities

**DOI:** 10.1111/ajr.70092

**Published:** 2025-09-22

**Authors:** Lauren Ware, Mitchell McGrath, Oliva Walsh, Ethan T. Cardwell, Jane Tomnay, Dave Evans, Anne‐Marie Kelly, Jason J. Ong, Jane S. Hocking, Fabian Y. S. Kong, Teralynn Ludwick

**Affiliations:** ^1^ Melbourne School of Population and Global Health University of Melbourne Parkville Victoria Australia; ^2^ Centre for Excellence in Rural Sexual Health University of Melbourne Parkville Victoria Australia; ^3^ Mat‐Su College University of Alaska Anchorage Palmer Alaska USA; ^4^ Melbourne Sexual Health Centre Carlton Victoria Australia; ^5^ School of Translational Medicine Monash University Melbourne Victoria Australia

**Keywords:** Australia, digital health service, qualitative study, STI screening, youth

## Abstract

**Objective:**

This study investigated local public health authorities' perspectives on implementing a new online STI testing service for young people in regional/rural Victoria, Australia.

**Setting:**

Study was conducted to support the design of *Test it*, a free, online STI testing service being developed for Victoria. After completing an online registration and sexual health questionnaire, users receive an electronic test request form to take directly to a pathology centre without seeing a general practitioner (GP).

**Participants:**

Ten individuals responsible for the coordination of community sexual health services within 7 Victorian Local Public Health Units (LPHUs) and the Department of Health, and a state‐wide health promotion organisation.

**Design:**

Semi‐structured interview topics covered: attitudes towards online STI testing, advantages and challenges of online STI testing for regional and rural communities, and strategies to support effective implementation.

**Results:**

Participants were enthusiastic about the potential of an online STI testing service, perceiving it to offer options in settings with limited sexual health providers, provide users with greater anonymity (bypassing GPs), improve efficiency, and support LPHUs to deliver their priorities. However, needing to attend a pathology service in‐person may reduce appeal and create barriers given limited transport/opening hours and stigmatisation concerns by young people. Navigating treatment, if needed, may also be challenging.

**Conclusion:**

Despite offering advantages, regional/rural young people are likely to encounter barriers to online STI testing that are different from and more significant than those faced by young people in urban areas. Strategies are needed to support implementation in regional/rural areas to realise the full potential of online testing to improve equitable access for underserved communities.

**Summary**

- What is already known on this subject?
  ○ Australian regional and rural young people are more likely than urban counterparts to prefer online STI testing, including for reasons of privacy and confidentiality.
  ○ Despite a preference for online testing, some studies show lower uptake of online STI testing among regional and rural users compared to metropolitan users.
  ○ There is a need to understand how to support the implementation of online STI testing in regional and rural settings to promote equitable uptake.
- What does this study add?
  ○ Public health authorities in regional and rural Victoria would value the addition of an online testing option, but identify various limitations associated with the use of a self‐navigated pathology model in regional and rural communities.
  ○ The study raises the need for targeted implementation strategies to address regional/rural‐specific barriers to use, including recommendations for sensitivity training for local pathology providers and care navigation support if users require treatment.

## 1 Introduction

Globally, rates of sexually transmitted infections (STIs) among young people aged 15 to 29 years are high and increasing, including in Australia [1, 2, 3]. However, young people face barriers to getting tested and treated for STIs, including lack of access to sexual and reproductive health services, stigmatisation, embarrassment and confidentiality concerns [4, 5, 6, 7]. These barriers are compounded in regional and rural areas, where specialist sexual and reproductive health services are limited, often with restricted opening hours and staffing due to resource constraints [8, 9]. In regional and rural settings, concerns around privacy and trust in local in‐person services have been identified by young people as key barriers to accessing sexual health services [6], with young people reluctant to talk to family doctors about sexual health issues [8, 9]. Further, young people may lack transport, time, or money to seek out more anonymous and free sexual health services in regional centres and cities [6, 10, 11, 12].

Online STI testing offers a solution to overcoming many of these barriers and to improving equitable access. Service models for online STI testing typically take one of three forms: (1) self‐testing, where users receive a test kit by mail, self‐collect specimens, and test and interpret their own results at home; (2) self‐sampling, where users receive a test kit by mail, self‐collect specimens at home, then return the kit by post or drop off to a pathology service for testing; (3) self‐navigated pathology, where online users receive an electronic pathology form and then attend an associated pathology service for sample collection [13, 14].

Studies show that online STI testing services increase testing rates, reduce time to testing, and are preferred by young people [15, 16, 17, 18, 19]. Recent studies from Australia find that rural young people are significantly more likely than their urban counterparts to prefer online STI testing, particularly due to privacy and confidentiality [15, 20]. However, the majority of STI testing in Australia occurs in general practices (GP clinics), especially in regional and rural Victoria [6, 21]. In recent years, the Victorian Government has invested in sexual and reproductive health hubs in regional areas as part of a women's health initiative [22]; however, many are staffed only part‐time, thus limiting capacity and access. While some online STI testing services are available, a recent service review shows that access to these services may be limited due to eligibility restrictions and cost, with some not meeting quality benchmarks [14].

While interest is high among rural youth, it remains unclear to what extent online STI testing is meeting their needs in practice, with some studies showing lower rates of uptake among rural youth compared to youth living in metropolitan areas [23, 24, 25]. This gap between high interest and low utilisation raises questions about the suitability of service design for implementation in regional and rural areas. In the context of designing a self‐navigated pathology service called ‘*Test it*’ to serve the state of Victoria, we interviewed local public health authorities to explore their views on the role of online STI testing for young people in regional and rural communities, the perceived benefits and limitations of a self‐navigated pathology model for use in these communities, and strategies for supporting implementation.

## 2 Materials and Methods

### 2.1 Setting

Victoria's nine LPHUs were established in 2020 as part of a decentralised public health response to manage local outbreaks of COVID‐19 in the state. Since 2022, their mandate has expanded to cover disease prevention and population health more generally, including the management and control of STIs and other notifiable diseases [26]. LPHUs manage programmes related to disease prevention and population health, namely contact tracing, partner notification for STIs, and case and outbreak management. They are tasked with developing strategic plans to implement the 4‐year Victorian Public Health and Wellbeing Plan, which includes improving equitable access to sexual and reproductive health [27]. LPHUs work closely with their local primary and community health services, other state government agencies, and local government and communities to deliver strategic priorities and manage control of infectious diseases; however, they do not provide any clinical services.

Located in metropolitan Melbourne, Melbourne Sexual Health Centre (MSHC) is the only specialist sexual health provider in Victoria that offers full‐time, free sexual healthcare, currently providing over 60 000 consultations a year [28]. To improve access to sexual health services throughout the state, the Victorian government has invested in several initiatives, including: (1) funding 11 new sexual and reproductive health hubs between 2017 and 2023 (time of this study), with 7 in regional areas (expanded to a total of 20 hubs in 2025, with 12 in regional areas); (2) a hub‐and‐spoke model in which the MSHC supports capacity building for 10 partner GP clinics located in outer Melbourne and regional areas [29]. Additionally, from 2017, the Department of Health has funded a Doctors in Secondary Schools initiative, where participating GPs and nurse practitioners provide health services to students in 100 disadvantaged Victorian secondary schools across the state [30]. While sexual health is not an explicit focus of the school programme, it serves as a key contact point for regional and rural youth with health services.

To improve access to STI testing, the MSHC, in collaboration with the University of Melbourne, is developing an online STI testing service (*Test it*) using a self‐navigated pathology model. The service model works as follows: eligible users (asymptomatic, Victorian residents, aged 16 years or older) begin by completing an online registration and sexual health questionnaire. Based on their responses, they receive an automatically generated test request form (to test for chlamydia, gonorrhoea, syphilis, and HIV) to take to a pathology provider of their choice. Those who test positive for chlamydia or gonorrhoea will receive automated referral letters to take to a provider of their choice for treatment, and those who test positive for HIV or syphilis will be managed by MSHC. Some features of the model have since been modified and will be reported in future papers.

This study is part of a collection of formative research supporting the design of *Test it* and its future implementation in regional and rural Victoria, generally covering Modified Monash classifications 2 to 5 (regional centres to small, rural towns). Other studies were conducted with young people in Victoria to inform service design and acceptability, and with health providers in regional and rural Victorian health services to explore clinical barriers to implementation.

### 2.2 Recruitment and Sampling

We invited public health authorities who are responsible for sexual health service delivery in regional and rural areas to participate in interviews, including: (1) representatives from eight Victorian LPHUs for whom we were provided with contacts from the Department of Health; (2) Department of Health staff whose portfolios covered sexual health; (3) a representative of a state‐wide Victorian sexual and reproductive health promotion organisation that is strongly connected with regional and rural service provision. Recruitment was conducted through purposive sampling, inviting individuals based on their roles within organisations involved in sexual and reproductive health service delivery in regional and rural areas. Participants were contacted by email between June and August 2023 and received two follow‐up emails spaced at least 1 week apart. One participant invited their colleague to participate in their interview, resulting in two individuals in one interview. Plain language statements and consent were provided electronically and signed prior to interviews.

### 2.3 Data Collection and Analysis

Semi‐structured interviews covered (S1):

- Attitudes towards an online STI testing service
- Advantages and challenges of an online self‐navigated STI testing service for young people in regional and rural communities
- Strategies to support effective implementation

Interviews were conducted virtually using Zoom video conferencing software by three members of the research team (O.W., M.M., T.L.) and were around 45 min in length. Interview participants selected locations they felt comfortable completing the interview, most commonly in private offices. Interviews were primarily conducted by T.L. and O.W. (both women); two were conducted by M.M., who is a man. T.L. (PhD) is the project manager and Research Fellow in the Sexual Health Unit at the University of Melbourne. As T.L. is a senior implementation researcher, research questions were oriented towards an implementation‐focused approach. O.W. (MPH) was a Research Assistant supporting the project, and M.M. was an MPH postgraduate student completing a research project component. Interviewers had no prior relationships with participants. Using a semi‐structured interview guide allowed interviewers to adapt the questionnaire and include new prompts for more in‐depth enquiry as topics emerged during the interviews. Interview audio recordings were transcribed using the automated service Otter.ai and reviewed for accuracy. Field notes were recorded during interviews and discussed as a team to support the identification of preliminary themes and codes. A qualitative content analysis was used to identify attitudes, concerns, barriers, facilitators, and strategies to support implementation in regional and rural communities. T.L., M.M. and L.W. collectively developed the preliminary coding structure based on a full reading of the transcripts and joint discussion. A hybrid analytic approach was used: codes were primarily established deductively according to the interview guide; however, some codes emerged inductively. L.W. conducted coding in NVivo 14 and would brief T.L. after coding every second interview for refinement of the codes. Each iteration of the coding framework was saved with comments outlining reasons for changes.

Ethical approval was obtained from the University of Melbourne (2023‐25358‐41317‐4). Identifying participant information, including names, organisations, and references to location, was removed during the transcribing of interviews, with each participant being allocated a code number. Participation was voluntary; participants could withdraw consent at any time. Recordings of interviews are stored securely at the University of Melbourne for 5 years from the date of publication.

## 3 Results

Ten individuals (9 women) participated in interviews, representing:

- 7 LPHUs (6 clinically trained Sexual and Reproductive Health Nurses, 1 Acting Clinical Director).
- Department of Health (2 Senior Policy Officers for sexual and reproductive health).
- 1 state‐wide Victorian sexual and reproductive health promotion organisation (Health Promotion Manager).

Four themes were identified in response to our three research questions. Participants' perspectives on the relevance and suitability of online STI testing for young people in regional and rural communities, including attitudes, service benefits, implementation challenges, and recommendations, are aligned with the research questions and presented below.

### 3.1 Research Question: What Is the Role of Online STI Testing for Young People in Regional and Rural Communities?

#### 3.1.1 Theme 1: Attitudes Towards Online STI Testing Services for Regional and Rural Communities

Overall, participants expressed positive views towards online STI testing services for regional and rural communities in Victoria, being ‘big advocate(s) of systems like this’ (ID5). They described it as a tool that ‘would solve a lot of our problems’ (ID8), noting the service's potential to ‘increase our testing rates and to increase awareness’ (ID9) and its alignment with LPHU strategic priorities. Given that LPHUs are responsible for delivering on strategic priorities in regional areas, largely through promoting service coordination, but without significant budgets for new services, the online service was valued for its potential to extend service reach without additional cost.

### 3.2 Research Question: What Are the Perceived Benefits and Limitations of a Self‐Navigated Pathology Model for Use in These Communities?

#### 3.2.1 Theme 2: Advantages of Implementing Online STI Testing in Regional and Rural Areas

Respondents felt that online STI testing would benefit regional and rural community members and healthcare providers alike.

##### 3.2.1.1 Expanding Access and Service Options

Key benefits for communities included overcoming access barriers, providing service options and overcoming limitations found in existing services. Respondents thought that an online STI testing service could ‘particularly in the regions… probably reach people who otherwise wouldn't access services’ (ID7) by expanding service reach in the context of geographic barriers and limited‐service providers.

Importantly, respondents indicated that an online STI testing service would provide more confidentiality to patients compared to traditional STI testing services. They perceived that a lack of anonymity in small towns (in which everyone knows everyone) was a key barrier to young people seeking STI testing:

It's really hard in regional and rural areas for young people to navigate services and to try to access services with… receptionists knowing them, or they know the GP (ID9)

Overall, the online service was seen as complimenting existing STI testing services, rather than replacing them. The overwhelming view among participants was that an online STI clinic would greatly benefit regional and rural communities by providing choice and options for STI testing:

What people want is options… so the more options you give, the less likely someone's going to find all of those options unpalatable if they really do want to get help. And I think an online service does provide a nice option (ID5)

It was also valued as an alternative and complement to self‐collected postal options and school‐based GP services. Participants raised several issues with postal services, including that ‘young people aren't posting anything, really, so they're not gonna know where their post boxes are’ (ID2). They also felt that ‘most young people that I would have spoken to that are getting tested wouldn't have wanted the kits to be posted to home’ due to privacy concerns (ID9).

While some schools have part‐time nurses and GPs available on‐campus, one respondent noted that the Doctors in Secondary Schools initiative is not ideal for STI testing:

A great initiative is Doctors in Secondary Schools, but, you know, often teachers are wondering where students are, you know, what are they doing? Who are they seeing?… and why aren't they in class? If they've been marked absent accidentally, you know, they go home, and mom and dad are asking questions… a really difficult system to navigate (ID9)

##### 3.2.1.2 Optimising In‐Person STI Services Through Streamlining Asymptomatic Screening

Beyond user benefits, respondents felt that an online STI testing service would generate efficiency gains for regional and rural service provision by reducing administrative burden (releasing time for care) and by creating an opportunity to streamline the asymptomatic screening process:

I think this will release time to care… it's about reducing administrative burden to allow clinicians to have face‐to‐face contact with patients rather than spending all of their time on the administrative side… allow[ing] clinicians to be able to work at their best with people: use the time that they have available to be able to do the things that computers can't do (ID2)

As such, an online service targeted towards asymptomatic testing would also help to reserve limited, in‐person appointments with regional and rural health professionals for more complex STI cases:

When you've got somebody who's a health professional who's skilled in sexual health, perhaps those skills are better targeted for people with a greater risk or people who are symptomatic (ID7)

#### 3.2.2 Theme 3: Challenges in Implementing Online STI Testing in Regional and Rural Areas

While participants saw many potential benefits, they all anticipated specific challenges associated with implementation in regional and rural areas.

##### 3.2.2.1 Lack of Prioritisation of STIs

At the health services level, one participant highlighted that where sexual and reproductive health services and resources do exist, they tend to be focused on pregnancy:

While STI is a part of it, a big focus of our work has been unplanned pregnancy, so access to medical and surgical termination for women in the [region] (ID8)

##### 3.2.2.2 Inadvertent Disclosures, Stigma and Consent

While many participants felt that online testing could potentially offer more anonymity in small towns, several indicated that young people are ‘concerned about breach(es) of confidentiality’ (ID7) and were worried that the fear of inadvertent disclosures might remain a barrier and ‘deterrent’ to young people testing at pathology centres (ID6):

And as I said, inadvertent disclosure again. When you're talking about rural and regional areas, you could be, you know, a neighbour, or a cousin, or someone who's actually at the pathology service, whether it be on reception, or as one of the people that are collecting the specimens. And I know in the past, there's also been concerns about even when the specimen gets to the lab: who's going to see this? Who's going to see my request? (ID10)

Further, young people need consent to leave school and may need to inform caregivers of their whereabouts. This can make it difficult for young people from unsupportive environments or with strict caregivers to access pathology services for STI testing.

##### 3.2.2.3 Logistical Barriers

A few participants indicated that the limited availability and opening hours of pathology clinics in regional and rural communities could act as a barrier for using the online STI testing service, including for students who would need to access them during school hours. Smaller communities ‘probably don't have standalone pathology collection places anyway’ (ID7), requiring users to find transport to a larger town to undertake testing. Further, young people who cannot drive themselves may be dependent on public transport to access services, which itself can be limited and time‐consuming in regional and rural areas:

Transport disadvantage is a big issue. Even within [large regional city]… if you lived on the other side of town, our public transport's not very great. So it'd be two buses, and they probably wouldn't line up very well. So, yeah, for a young person who doesn't drive, even accessing our services can be challenging (ID8)

##### 3.2.2.4 Reaching Disadvantaged Populations

Participants raised numerous concerns about who might be left out from accessing the service in regional and rural areas, including disadvantaged youth, those without Medicare, non‐English speakers, and those living on farms without internet access. They raised the issue that disadvantaged youth, who would most benefit from comprehensive in‐person services, including referrals to other resources and supports, may be disadvantaged by the shift to online services:

I feel that we capture those young people that aren't disadvantaged really… they know how to access services. Usually they've got supportive parents at home. But it's those ones that are disadvantaged, that haven't got that support: how do we capture that in these online tests and look at all those kind of issues that they might have? (ID9)

As the service currently requires individuals to hold a Medicare card or pay out‐of‐pocket, they were concerned that key groups who would benefit from access to online testing would be excluded, particularly international students, asylum seekers and refugees, and immigrants who are part of regional and rural communities:

We know what the risk profiles are of people who are ineligible for Medicare. And I would really worry about missing a number of groups of people who are in desperate need of a service like this, particularly international students and asylum seekers and refugees who have higher concerns around confidentiality. So this kind of service would be really appropriate for them because it's more accessible and more private. So issues around cost for those groups are huge (ID1)

##### 3.2.2.5 Treatment Pathways

Respondents identified several challenges in establishing treatment pathways in regional and rural areas for those who test positive using the online STI testing service. While good in theory, logistical barriers raised questions about whether online STI testing remains advantageous once users require treatment:

I think it's a great option and a great opportunity to increase our testing rates and to increase awareness… but yeah, what do you do when you've got that positive test? How would we get that treatment out there? (ID9)

One participant was uncertain if an online service would effectively improve testing uptake if young people were not already engaged with the health system:

Which again, comes back to why didn't they go to the local provider in the first place? Are they likely to actually go in now and see them for care? Or are they able to even see them? Do they have a local provider? (ID10)

Participants raised key issues related to administering prescriptions. They noted that options for treatment are ‘really limited’ outside of regional cities, and most rural GPs are likely to charge an out‐of‐pocket fee (ID8). While nurse‐led models are common for sexual and reproductive health care in regional and rural areas and were perceived as ‘an effective approach to this kind of work’ (ID2), most nurses lack prescribing authority, which can create potential bottlenecks in providing treatment to patients.

They also conjectured that some GPs, particularly those from private regional and rural clinics, may be resistant to receiving referrals from online services which may be perceived as less ‘legitimate’ (ID8):

They're not going to be everybody's cup of tea. And you probably will get some clinicians who won't agree with it… some of them are really even not keen on walk‐in services (ID7)

Additionally, a participant expressed that GP clinics may choose not to advertise sexual and reproductive health services (including the online STI testing service) out of concern that they will be unable to meet demand of new patients. They were concerned about further ‘overburdening’ clinicians who are ‘personally passionate about sexual reproductive health’, by taking on more patients (ID2).

##### 3.2.2.6 Acceptability of the Service in the Community

Some participants were unsure how visible the service could and should be in regional and rural communities, given conservative attitudes and possible backlash:

A more conservative voter does tend to live further out in the rural regional spaces, and then that is often coupled with more conservative views around sexual reproductive health (ID2)

While some participants felt these attitudes primarily focused on anti‐abortion views, others still ‘expect there probably is some resistance’ as a more accessible online service could be perceived by community members as ‘promoting young people to be promiscuous, because, you know, you're normalising SRH [sexual and reproductive health] screening. Well, you're normalising sex’ (ID7). Further, conservative attitudes in regional and rural areas, including conscientious objectors, may deter health providers from letting patients know that they provide STI treatment:

A lot of GPs will provide medical abortion or will, you know, do testing and treatment for STIs, but they won't include anything on their website. They won't list 1800 My Options [a pro‐choice statewide service that provides information on sexual and reproductive health services]… because of fear… you know, the backlash that's happening now around… athat conversation around sexual and reproductive health (ID2)

### 3.3 Research Question: What Strategies Could Support Implementation?

#### 3.3.1 Theme 4: Recommendations for the Service

In response to the limitations raised, participants offered recommendations for service inclusions and promotional strategies to better engage regional and rural communities in online STI testing.

##### 3.3.1.1 Resources and Training

Given people's lack of familiarity with online testing and to ‘alleviate some of that… fear of the unknown’ (ID2), they recommended that additional resources be included, such as plain English guides on how to use the service, resources on other sexual and reproductive health issues (e.g., information about contraception, consent and sexual assault), and information about supporting services and hotlines.

One participant recommended that training be provided to pathology services, including front‐of‐house staff, to avoid potential stigmatisation of individuals resulting from lack of sensitivity around gender, sex and sexuality:

I've seen a number of receptionists really like “hate crime” [used casually to describe harm] people. And not with ill intention, but you know, mis‐gender people… or make people overly explain in a public place why they're here… providing that education to some of the pathology services… something that could say “this is happening, we've got this new digital service, here's a link to a basic sex ed 101”… and then always some stuff on what the difference [is] between gender and sex, gender identity, sexuality (ID2)

##### 3.3.1.2 Expand Eligibility and Range of Tests Offered

To provide more comprehensive care and reach more people, there were recommendations to allow ‘even uncomplicated symptoms [to test] online’ (ID8). Suggesting that ‘half the time they're just going to be chlamydia and gonorrhoea’, allowing those with uncomplicated symptoms to use the service would remove ‘just another barrier’ to testing easily treatable STIs that are imposed by strict, asymptomatic eligibility (ID8). Participants also recommended including Hepatitis C testing within the online service to align with the Victorian Government's goal of disease eradication, and to provide more ‘synergy and holistic care’ to users (ID4).

##### 3.3.1.3 Connecting With Local Resources and Promotion

Multiple participants suggested that LPHUs could support the service by serving as ‘care navigators’ (ID6) that connect users with local healthcare providers:

Say somebody got diagnosed with gonorrhoea or syphilis, and needed treatment… you could contact the care navigator. Who would find out, you know, where does the person live? Where do you get a prescription? Where do you fill it? And who's going to give it? And we could develop a network of people who could do that (ID6)

Participants urged for promotion strategies to be targeted and catered to individual regional and rural communities, highlighting that each community has different needs and perspectives. Turnover of staff is an ongoing issue in regional and rural clinics which may pose a challenge for the promotion of the online STI testing service to the community. They highlighted a need to have ongoing education with regional and rural health services to enable ‘ongoing, constant promotion’ which will ‘constantly keep reminding people of the service’ (ID8).

## 4 Discussion

Our study is one of the few to focus on the implementation of online STI testing services in regional and rural communities [31, 32, 33] and offers valuable insight into how well a self‐navigated service might overcome barriers to testing and persistent challenges that are unlikely to be addressed. We found that LPHUs were enthusiastic about the potential of an online STI testing service to serve regional and rural Victoria. They perceived it as a valuable addition to locally available services by providing choice and options, supporting improved anonymity, and enabling users to bypass sexual health consultations with GPs, which are both undesired by young people and difficult to obtain, given GP shortages in regional and rural communities. At the same time, several factors may make the service less appealing to young people and operate less optimally in regional and rural locations compared with metropolitan areas. The requirement to attend a pathology service still presents issues for patient confidentiality in small communities, the potential for stigmatisation, and transport barriers for young people. Further, users who test positive using the online service will nevertheless have to navigate local health services where STI treatment options may be limited.

Though likely to work imperfectly for regional and rural communities, a self‐navigated model for online STI testing offers different advantages compared with traditional clinic‐based services and other alternative models, including collecting self‐sampling kits via post or vending machine. Studies from Australia and elsewhere have shown that return rates of self‐sampling kits are often low (< 60%) [32, 33, 34]. While there are various reasons for low return rates, distance to post offices and lack of familiarity and/or interest in using the post, as identified in this study, could further compound low return rates among young people. While vending machines offer an anonymous, place‐based option for users to access self‐sampling kits, they have more limited reach and require local approvals [35]. Importantly, the online self‐navigated model presents no significant implementation costs for LPHUs or local councils, with Victoria's specialist service (MSHC) absorbing the running costs. While all options have their own unique advantages and disadvantages, no single model is able to overcome the full range of barriers to accessing sexual health services encountered in regional and rural Victoria. As such, providing options and choice, as highlighted in our study findings, is of key value.

Our study identified several strategies to offset anticipated barriers to use of an online, self‐navigated STI testing service in regional and rural areas, particularly around treatment pathways. Users who test positive for gonorrhoea or syphilis require injectable antibiotics that must be administered by health professionals. LPHUs suggested they could serve as care navigators to connect online users with local providers for treatment. Recognising that a subset of clinicians may be uninterested in receiving referrals from an online service [36] or perceive them as less legitimate, LPHUs can help connect users with amenable providers, including the newly funded state sexual and reproductive health hubs and regionally based partner clinics of MSHC [31]. Though not addressed in this study, providing telehealth and electronic prescriptions for uncomplicated cases of chlamydia, as offered in some online services, could also reduce barriers to care [31]. Additionally, providing training for local pathology services, including front‐of‐house staff, on destigmatising STIs and inclusive ways to serve LGBTIQ+ clients could help prevent negative experiences which could deter young people from testing [37, 38].

To be successful, promotion is needed to generate awareness of the new service. While promotion via local media and organisations is often key in regional and rural communities [39], there may be a more limited role in the case of online STI testing, given concerns raised in this study about possible backlash among conservative communities. Digital promotion uses targeted approaches which are likely to better reach youth searching for STI testing services [40], and may also prevent public backlash by tailoring who sees the content.

While improving access and equity generally, a self‐navigated online STI testing service model may reinforce disadvantage for those living on farms without internet connections, those without Medicare, and communities where pathology services are geographically distant. Online alternatives need to work in conjunction with greater investments in regional and rural health services and infrastructure. Further, there is the need to raise the profile of sexual health in regional and rural contexts, where the focus on unintended pregnancy tends to overshadow attention on STIs by both the community and health services.

## 5 Limitations

While our sample size was small, our study achieved high representation of LPHUs across the state, drawing upon key informants with strong knowledge of the local health and sexual health service landscape and connection to communities. While LPHUs are well positioned to voice implementation considerations related to the health service landscape, further qualitative research with young people would allow them to voice access‐related barriers from a youth perspective and will build on our existing quantitative data collection. Future research should also assess what types of local promotion may be most suitable to raising awareness without generating opposition and explore the appropriateness and acceptability of this service model for young Aboriginal and Torres Strait Islander peoples and those from culturally and linguistically diverse backgrounds.

## 6 Conclusion

Online STI testing services are known to improve access and are particularly appealing to young people. While young people in regional and rural areas are even more likely than their urban counterparts to prefer online testing, they may encounter barriers to using online testing that are different from, and more significant than, those faced by users in urban areas. Strategies are needed to support implementation in regional and rural areas to fully capitalise on the potential of online testing models to improve equitable access in these underserved communities.

## Author Contributions

**Lauren Ware:** writing – original draft (lead), formal analysis (lead), writing – review and editing. **Mitchell McGrath:** investigation, formal analysis (supporting), writing – review and editing. **Oliva Walsh:** project administration, investigation. **Ethan T. Cardwell:** writing – review and editing. **Jane Tomnay:** writing – review and editing. **Dave Evans:** writing – review and editing. **Anne‐Marie Kelly:** writing – review and editing. **Jason J. Ong:** writing – review and editing. **Jane S. Hocking:** conceptualization (supporting), funding acquisition, writing – review and editing. **Fabian Y. S. Kong:** writing – review and editing, supervision. **Teralynn Ludwick:** conceptualization (lead), writing – original draft (supporting), investigation, methodology, supervision, writing – review and editing.

## Ethics Statement

Ethical approval was obtained from the University of Melbourne (2023‐25358‐41317‐4).

## Conflicts of Interest

The authors declare no conflicts of interest.

## Supporting information


**Data S1:** ajr70092‐sup‐0001‐Supinfo.docx.

**Data S2:** ajr70092‐sup‐0002‐Supinfo.docx.

## Data Availability

Data available on request from the authors.
