# Supplementary material for: Implementing an Online Sexually Transmitted Infection Testing Service for Young People in Regional and Rural Victoria, Australia: Insights From Local Public Health Authorities
Source: Aust J Rural Health. 2025 Sep 22;33(5):e70092. doi: 10.1111/ajr.70092 (PMC12451655; doi:10.1111/ajr.70092)
Supplement: Supplementary file 1 — Data S1: ajr70092‐sup‐0001‐Supinfo.docx. [file AJR-33-0-s001.docx]

**Supplemental materials file S2: COREQ (COnsolidated criteria for REporting Qualitative research) Checklist**

| **Topic** | **Item No.** | **Guide Questions/Description** | **Response** |
| --- | --- | --- | --- |
| **Domain 1: Research team and reflexivity** |  |  |  |
| *Personal characteristics* |  |  |  |
| Interviewer/facilitator | 1 | Which author/s conducted the interview or focus group? | OW, MM and TL |
| Credentials | 2 | What were the researcher’s credentials? E.g. PhD, MD | TL holds a PhD  OW holds an MPH  MM holds an MPH |
| Occupation | 3 | What was their occupation at the time of the study? | TL is a Research Fellow in the Sexual Health Unit at the University of Melbourne.  OW was a Research Assistant that supports the team.  MM was an MPH postgraduate student completing a research project component. |
| Gender | 4 | Was the researcher male or female? | TL and OW are women, MM is a man. |
| Experience and training | 5 | What experience or training did the researcher have? | TL has published numerous qualitative  studies. OW and MM have completed qualitative  training as part of their MPH studies and received  guidance on interviewing by TL. |
| *Relationship with participants* |  |  |  |
| Relationship established | 6 | Was a relationship established prior to study commencement? | The participants did not have a prior relationship  with the interviewers. |
| Participant knowledge of the interviewer | 7 | What did the participants know about the researcher? e.g. personal goals, reasons for doing the research | Participants were told that the interviewers were part of a research team working with the  Melbourne Sexual Health Centre to design and implement and online STI testing service in Victoria, Australia. |
| Interviewer characteristics | 8 | What characteristics were reported about the inter viewer/facilitator? e.g. Bias, assumptions, reasons and interests in the research topic | The manuscript reports that this study was conducted as part of a larger effort to design and implement and online STI testing service in Victoria, Australia. |
| **Domain 2: Study design** |  |  |  |
| *Theoretical framework* |  |  |  |
| Methodological orientation and Theory | 9 | What methodological orientation was stated to underpin the study? e.g.  grounded theory, discourse analysis, ethnography, phenomenology, content analysis | Content analysis |
| *Participant selection* |  |  |  |
| Sampling | 10 | How were participants selected? e.g. purposive, convenience, consecutive, snowball | We used purposive sampling to select individuals working within LPHUs, the Department of Health and a state-wide Victorian sexual and reproductive health promotion organisation working in sexual and reproductive health in regional areas where services are more limited. They were purposively selected based on our aims of understanding local public health authorities’ and community perspectives to build equitable and acceptable sexual health services in these areas. |
| Method of approach | 11 | How were participants approached? e.g. face-to-face, telephone, mail, email | Invited by email |
| Sample size | 12 | How many participants were in the study? | 10 |
| Non-participation | 13 | How many people refused to participate or dropped out? Reasons? | No one dropped out. 1 LPHU did not respond to our invitation to participate. |
| *Setting* |  |  |  |
| Setting of data collection | 14 | Where was the data collected? e.g. home, clinic, workplace | Interviews took place online |
| Presence of nonparticipants | 15 | Was anyone else present besides the participants and researchers? | No |
| Description of sample | 16 | What are the important characteristics of the sample? e.g. demographic data, date | Individuals responsible for sexual health services within Victorian Local Public Health Units (LPHUs)/Department of Health, and a state-wide health promotion organisation where service catchments include regional and rural areas. Almost all participants were women, and over half were clinically trained Sexual and Reproductive Health Nurses. |
| *Data collection* |  |  |  |
| Interview guide | 17 | Were questions, prompts, guides provided by the authors? Was it pilot tested? | The interview guide was not provided in advance.  However, the PLS provided an overview of the types of questions that would be asked. We tailored questions to be relevant to the situation of the respondent. |
| Repeat interviews | 18 | Were repeat interviews carried out? If yes, how many? | No |
| Audio/visual recording | 19 | Did the research use audio or visual recording to collect the data? | Interviews were audio and video recorded . |
| Field notes | 20 | Were field notes made during and/or after the interview or focus group? | A set of field notes were collected and discussed  after each interview. |
| Duration | 21 | What was the duration of the inter views or focus group? | Interviews were about 45 minutes long |
| Data saturation | 22 | Was data saturation discussed? | The sample size was limited by the number of  LPHUs in the state and the number of individuals responsible for sexual health services within the Department of Health. |
| Transcripts returned | 23 | Were transcripts returned to participants for comment and/or correction? | No |
| **Domain 3: analysis and findings** |  |  |  |
| *Data analysis* |  |  |  |
| Number of data coders | 24 | How many data coders coded the data? | LW coded the data with further refinement of the codes by TL. |
| Description of the coding tree | 25 | Did authors provide a description of the coding tree? | Yes |
| Derivation of themes | 26 | Were themes identified in advance or derived from the data? | Derived from the data |
| Software | 27 | What software, if applicable, was used to manage the data? | Nvivo 14 was used to code the data . |
| Participant checking | 28 | Did participants provide feedback on the findings? | No |
| *Reporting* |  |  |  |
| Quotations presented | 29 | Were participant quotations presented to illustrate the themes/findings?  Was each quotation identified? e.g. participant number | Yes |
| Data and findings consistent | 30 | Was there consistency between the data presented and the findings? | Yes |
| Clarity of major themes | 31 | Were major themes clearly presented in the findings? | Yes |
| Clarity of minor themes | 32 | Is there a description of diverse cases or discussion of minor themes? | Yes |
