# Supplementary material for: Implementing an Online Sexually Transmitted Infection Testing Service for Young People in Regional and Rural Victoria, Australia: Insights From Local Public Health Authorities
Source: Aust J Rural Health. 2025 Sep 22;33(5):e70092. doi: 10.1111/ajr.70092 (PMC12451655; doi:10.1111/ajr.70092)
Supplement: Supplementary file 2 — Data S2: ajr70092‐sup‐0002‐Supinfo.docx. [file AJR-33-0-s002.docx]

**Supplemental materials file S1: Interview Guide**

**Topic: Warm-up**

1. To start off with, can you tell me a bit about your role in supporting sexual health (SH) services, and sexually transmitted infection (STI) testing services specifically?
2. In providing STI testing services in your region, what do you perceive as the biggest challenges?

**Topic: Strategic priorities**

1. Can you tell me about some of the strategic plans and priorities for SH services in your region?
   1. Any focus on digital services?
   2. What are some of the key programs that are being implemented? What role does the Local Public Health Unit (LPHU)/your organisation have?
2. When you first heard about the online STI testing service, what was your initial impression?
   1. Are there ways in which you think the online STI testing service could help achieve some of your SH priorities?
   2. Do you have any reservations?

**Provide overview of how the online STI testing service works**

**Topic: Access to STI testing**

1. Young people in rural areas may have difficulty getting to or using pathology services for testing.
   1. What do you think are the main barriers for young people to test at pathology services in rural Victoria?
   2. How can we make it more accessible for young, rural people to access local pathology providers for in-person testing?
   3. Does your organisation have any capacity to provide transportation support or other types of support to encourage testing?

**Topic: Access to STI treatment**

1. For young people with a positive test result, they will need treatment. If they need injections, they’ll need to be connected to local providers. What do you see as potential ways that the online STI testing service could work with local service providers?
   1. Do you have any reservations? What would be the challenges of implementing (prompt: Symptomatic referrals, treatment)?
   2. What issues are there with regards to capacity to provide injections (stock, staff etc)
2. What do you think the role of LPHUs could be with regards to engaging and promoting the online service?
   1. What stakeholders/services do you think would be important to engage when setting up the online STI testing service in your area? Why?
      1. Prompts: Doctors in Schools program, Public Health Networks

**Topic: Community attitudes**

1. In general, what has been the community’s response to sexual health programs and services in your area?
   1. How has the local council responded to positive or negative feedback from the community?
   2. How do you think your community might respond if this service was promoted in your area?

**Topic: Promotion recommendations**

1. What approaches do you think would work best for promoting the online STI testing service?
   1. Among the community?
   2. Among service providers?
   3. Do you think this service is something your LPHU/organisation would be interested in actively promoting? Why or why not?

**Topic: Wrap up**

1. Do you have any other comments or thoughts on how the online STI testing service could work in your region?
2. Is there anyone else you think we should speak to?
